# FZD7 Inhibitor SRI Attenuates High Glucose‐Induced Retinal Pigment Epithelial Cell Injury Accompanied by Ferroptosis‐Associated Changes via Suppression of the Wnt/β‐Catenin Pathway

**DOI:** 10.1002/edm2.70290

**Published:** 2026-07-22

**Authors:** Jiaojiao Jiang, Liu Zheng, Liwu Tan, Zhixiang Ding

**Affiliations:** ^1^ Department of Ophthalmology, the First Affiliated Hospital of Guilin Medical University Guilin Medical University Guilin China; ^2^ Department of Cardiovascular Nanxishan Hospital of Guangxi Zhuang Autonomous Region Guilin China

**Keywords:** diabetic retinopathy, ferroptosis, FZD7, retinal pigment epithelial cells, Wnt/β‐catenin pathway

## Abstract

**Purpose:**

This study investigated the protective effects of the Frizzled‐7 (FZD7) inhibitor SRI against high glucose–induced injury in human retinal pigment epithelial (ARPE‐19) cells. It also explored the underlying mechanism, focusing on whether SRI attenuates ferroptosis and apoptosis by inhibiting the Wnt/β‐catenin signalling pathway.

**Methods:**

ARPE‐19 cells were exposed to high glucose (25 mM) and treated with a non‐cytotoxic concentration of SRI (2 μmol/L), as determined by a CCK‐8 assay. Wnt/β‐catenin signalling was evaluated by measuring β‐catenin expression and phosphorylation. The expression of GPX4, DHODH, and FSP1, together with intracellular Fe²⁺, ROS, and MDA levels, was assessed to evaluate ferroptosis and oxidative stress. Apoptosis was analysed by examining Bax and Bcl‐2 expression, whereas mitochondrial ultrastructure was evaluated using quantitative transmission electron microscopy.

**Results:**

Under hyperglycaemic conditions, SRI suppressed Wnt/β‐catenin signalling by reducing β‐catenin expression and promoting its phosphorylation. SRI activated a GPX4‐independent ferroptosis defence pathway, evidenced by increased DHODH and FSP1 expression without affecting GPX4 levels. Furthermore, SRI reduced intracellular Fe²⁺, ROS and MDA accumulation, downregulated Bax, upregulated Bcl‐2 and improved mitochondrial morphology with partial restoration of cristae integrity. Collectively, these findings demonstrate that SRI alleviates high glucose‐induced oxidative stress, ferroptosis, apoptosis and mitochondrial damage.

**Conclusions:**

SRI protects ARPE‐19 cells from high glucose–induced injury by reducing ferroptosis‐associated oxidative stress, apoptosis and mitochondrial dysfunction. These protective effects are mediated, at least in part, through inhibition of Wnt/β‐catenin signalling and activation of a DHODH/FSP1‐dependent but GPX4‐independent ferroptosis defence pathway. These findings suggest that FZD7 represents a promising therapeutic target for diabetic retinopathy.

## Graphical abstract

SRI inhibits FZD7/Wnt/β‐catenin to upregulate DHODH/FSP1, alleviating ferroptosis, oxidative stress and mitochondrial damage against high glucose‐induced ARPE‐19 injury.

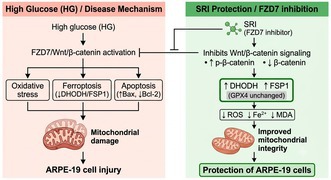

## 1 Introduction

Diabetic retinopathy (DR) is a common microvascular complication of diabetes mellitus and remains the leading cause of blindness among working‐age populations worldwide. Current estimates indicate that approximately 103 million individuals with diabetes have some degree of DR, and nearly 28 million develop vision‐threatening stages. This condition imposes a substantial burden on both quality of life and global healthcare systems [1]. The pathogenesis of DR is complex and multifactorial, involving a series of interconnected molecular and cellular events triggered by chronic hyperglycemia [2]. Key pathogenic mechanisms include oxidative stress, inflammatory activation, mitochondrial dysfunction, endoplasmic reticulum stress and advanced glycation end‐product accumulation [3], all of which contribute to retinal microvascular injury, neurodegeneration and pathological neovascularisation. Prolonged hyperglycemia also stimulates the production of pro‐inflammatory cytokines, including IL‐1β, thereby disrupting the inner blood‐retinal barrier (iBRB), which is maintained by the retinal neurovascular unit (NVU) comprising endothelial cells, pericytes, glial cells and neurons. This barrier dysfunction represents a critical early event in the progression of DR [4]. Furthermore, the neurovascular unit, comprising neurons, glial cells and vascular cells, plays a central role in maintaining retinal homeostasis and dysfunction of any component can adversely affect the entire retinal microenvironment [5]. Together, these pathogenic processes drive progressive retinal damage and ultimately lead to vision loss.

Recent studies have increasingly highlighted retinal pigment epithelium (RPE) dysfunction as a critical contributor to the progression of DR [6]. Located between the photoreceptors and the choroidal vasculature, RPE cells maintain blood‐retinal barrier integrity, regulate selective permeability, phagocytose photoreceptor outer segments and provide antioxidant protection essential for retinal homeostasis [7]. Under persistent hyperglycemic conditions, RPE cells undergo oxidative stress, mitochondrial dysfunction, lipid peroxidation and multiple forms of programmed cell death, including apoptosis, ferroptosis and pyroptosis. These pathological changes disrupt barrier integrity, promote vascular leakage and inflammation and ultimately accelerate DR progression [8]. Mechanistically, high glucose activates several pathogenic signalling pathways, including SP1/CFL2/AMPK/mTOR, KLF10/PERK/eIF2α/ATF4/CHOP and RACK1/PKC‐ε/ROS, all of which contribute to RPE dysfunction under diabetic conditions [9, 10, 11]. However, whether upstream Wnt/FZD7 signalling participates in regulating ferroptosis‐associated changes in diabetic RPE cells remains largely unknown. Therefore, investigating the role of FZD7 inhibition in this process may provide new mechanistic insights and potential therapeutic strategies for DR.

Among the various mechanisms of cell death involved in DR, ferroptosis has recently garnered considerable attention. Ferroptosis is a distinct iron‐dependent form of programmed cell death, primarily characterised by excessive lipid peroxidation and inactivation of glutathione peroxidase 4 (GPX4) [12, 13]. Accumulating evidence suggests that ferroptosis plays a critical role in DR pathogenesis. Specifically, in RPE cells exposed to high glucose, intracellular iron accumulation and lipid peroxidation are markedly increased, thereby triggering ferroptotic cell death [14]. Mechanistically, GPX4 serves as a central regulator of ferroptosis by detoxifying lipid peroxides in a glutathione‐dependent manner, thus preserving cellular integrity [15]. Importantly, recent studies have identified additional GPX4‐independent defence systems against ferroptosis. For instance, dihydroorotate dehydrogenase (DHODH) reduces ubiquinone (CoQ10) to ubiquinol (CoQ10H_2_), a potent lipid‐soluble antioxidant that mitigates lipid peroxidation [16, 17]. Similarly, ferroptosis suppressor protein 1 (FSP1) functions as an alternative protective pathway, further reinforcing cellular resistance to ferroptotic damage. Nevertheless, despite these advances, the regulatory mechanisms governing ferroptosis in RPE cells under hyperglycemic stress remain incompletely understood. In particular, the upstream signalling events and the interplay between GPX4‐dependent and independent pathways require further elucidation. Addressing these gaps may provide critical insights into DR pathogenesis and facilitate the development of targeted therapeutic interventions.

The Wnt/β‐catenin signalling pathway is an evolutionarily conserved regulatory system that governs cell proliferation, differentiation, apoptosis and survival [18]. Dysregulation of this pathway has been implicated in multiple retinal disorders, including DR and age‐related macular degeneration (AMD), highlighting its critical role in ocular pathophysiology [19]. Within this cascade, Frizzled (FZD) receptors act as key mediators by binding Wnt ligands and initiating downstream signalling [20]. Among them, FZD7 has recently attracted attention for its potential involvement in ferroptosis regulation. Evidence from oncology suggests that FZD7 modulates ferroptosis in a context‐dependent manner. In ovarian cancer, FZD7 suppresses ferroptosis and promotes chemoresistance through the β‐catenin/TP63/GPX4 axis [21]. By contrast, in gastric cancer, BCL6 enhances ferroptosis and inhibits tumour progression by transcriptionally repressing FZD7 within the same pathway [22]. Despite these insights, it remains unclear whether a similar regulatory mechanism operates in RPE cells under diabetic conditions. Beyond ferroptosis, FZD7 also plays a critical role in retinal angiogenesis. Studies using murine oxygen‐induced retinopathy (OIR) models have shown that FZD7 expression is markedly upregulated. Moreover, intravitreal administration of anti‐FZD7 neutralising antibodies or the soluble extracellular cysteine‐rich domain (CRD) significantly suppresses pathological retinal neovascularisation [23]. These findings underscore the importance of the FZD7/β‐catenin axis in pathological angiogenesis. SRI‐37892 (SRI) has emerged as a promising small‐molecule inhibitor that selectively targets FZD7. It competitively binds to the transmembrane domain (TMD) of FZD7 and blocks activation of the canonical Wnt/β‐catenin pathway [24]. Therefore, FZD7 represents a compelling therapeutic target for ischemic retinopathies and SRI provides a useful pharmacological tool to investigate FZD7‐mediated functions in the retina.

Despite its established role in angiogenesis, the potential protective effects of FZD7 inhibition in DR, particularly in relation to ferroptosis and OS, remain largely unexplored. Addressing this gap is critical for advancing the understanding of DR pathophysiology and for developing targeted therapeutic strategies. Furthermore, while FZD7 has been implicated in retinal angiogenesis in ischemic retinopathy models [23], its role in RPE cell survival and ferroptosis regulation under hyperglycemic conditions has not been previously explored. Based on these observations, we hypothesise that SRI attenuates ferroptosis and OS‐induced cellular injury in RPE cells under hyperglycemic conditions by inhibiting activation of the canonical Wnt/β‐catenin signalling pathway. Accordingly, this study aims to evaluate the protective effects of SRI in high glucose‐stimulated RPE cells and to elucidate the underlying molecular mechanisms. Collectively, this work seeks to provide mechanistic insights into FZD7‐mediated regulation in DR and to establish a foundation for the development of targeted therapeutic strategies to preserve retinal function and delay disease progression.

## 2 Methods

### 2.1 Cell Culture

ARPE‐19 cells were obtained from the Cell Resource Center of the Shanghai Institute of Life Sciences, Chinese Academy of Sciences and cultured in DMEM/F12 medium (Gibco, USA) supplemented with 10% fetal bovine serum (FBS) and 1% penicillin–streptomycin. Cells at passages 3–6 were randomly assigned to four experimental groups: normal glucose (NG, 5.5 mM glucose), high glucose (HG, 25 mM glucose), high glucose with vehicle control (HG + DMSO) and high glucose with SRI treatment (HG + SRI). Depending on the specific assay, cells were incubated for 24, 48 or 72 h before subsequent analyses, as described in the corresponding sections below.

### 2.2 Cell Viability Assay

ARPE‐19 cells were seeded into 96‐well plates at a density of 5 × 10^3^ cells/mL and incubated for 24 h to allow cell adhesion. Cells were then treated with SRI at concentrations of 0.5, 2, 8 and 16 μmol/L for 24, 48 or 72 h. Following treatment, 10% CCK‐8 solution (Glpbio, GK10001, USA) was added, and the cells were incubated at 37°C for an additional 2 h. Absorbance at 450 nm was subsequently measured using a microplate reader to assess cell viability.

### 2.3 Western Blot (WB)

Total cellular proteins were extracted using RIPA lysis buffer (Solarbio, Beijing) following 48 h of treatment. Protein concentrations were quantified using a BCA assay kit (Solarbio, Beijing). Equal amounts of protein (20 μg per lane) were resolved by 10% SDS‐PAGE and then transferred onto PVDF membranes. Membranes were incubated overnight at 4°C with primary antibodies against β‐actin (1:4000, 66009‐1‐Ig), FZD7 (1:1000, 16974‐1‐AP), β‐catenin (1:5000, 66379‐1‐Ig), phosphorylated β‐catenin (p‐β‐catenin; 1:5000, 80067‐1‐RR), VEGF (1:2000, 19003‐1‐AP), GPX4 (1:1000, 30388‐1‐AP), DHODH (1:5000, 14877‐1‐AP) and FSP1 (1:3000, 20886‐1‐AP) (all from Proteintech). After washing with TBST, membranes were incubated with species‐appropriate secondary antibodies for 1 h at room temperature. Protein bands were detected using an enhanced chemiluminescence reagent (Yasei, Shanghai) and quantified using ImageJ software.

### 2.4 Detection of OS Markers and Iron Content

Cells were harvested after 48 h of treatment for biochemical analyses. Malondialdehyde (MDA) levels were measured using an MDA assay kit (Elabscience, E‐BC‐K028‐M‐96T), and intracellular Fe^2+^ concentrations were determined using an iron assay kit (Purely, E1046). Intracellular reactive oxygen species (ROS) levels were assessed using the fluorescent probe dihydroethidium (DHE; Solarbio, CA1420). Cells were incubated with DHE (10 μmol/L) at 37°C in the dark for 30 min, followed by washing with phosphate‐buffered saline. Fluorescence images were acquired under identical exposure conditions and quantified using ImageJ software.

### 2.5 Transmission Electron Microscopy (TEM) for Mitochondrial Ultrastructure

After 48 h of treatment, cells were fixed in 2.5% glutaraldehyde at 4°C for 2 h, dehydrated through a graded ethanol series, embedded in epoxy resin and sectioned into 100‐nm ultrathin sections using a Leica ultramicrotome. The sections were subsequently stained with uranyl acetate and lead citrate and examined using a Hitachi transmission electron microscope. Mitochondrial ultrastructure was quantitatively evaluated in at least 50 mitochondria from 10 randomly selected fields per group. Image analysis was independently performed by two investigators blinded to the experimental conditions. Mitochondrial morphology was assessed using a semi‐quantitative scoring system (1–4), defined as follows: score 1, normal elongated or oval mitochondria with intact cristae and outer membranes; score 2, mild swelling with partial cristae disorganisation; score 3, marked swelling with cristae loss and increased membrane electron density; and score 4, severe shrinkage or vacuolisation with complete cristae disruption.

### 2.6 Statistical Analysis

Statistical analyses were performed using GraphPad Prism. All quantitative data are presented as mean ± standard error of the mean (SEM). For comparisons between two groups, unpaired *t*‐tests were applied, whereas differences among multiple groups were assessed using one‐way analysis of variance (ANOVA) followed by Tukey's post hoc test. A value of *p* < 0.05 was considered statistically significant. Significance levels are denoted as follows: *p* < 0.05, *p* < 0.01, *p* < 0.001 and *p* < 0.0001 versus the NG group; #*p* < 0.05, ##*p* < 0.01, ###*p* < 0.001 and ####*p* < 0.0001 versus the HG group.

## 3 Results

### 3.1 Effect of SRI on ARPE‐19 Cell Viability

**FIGURE 1:**
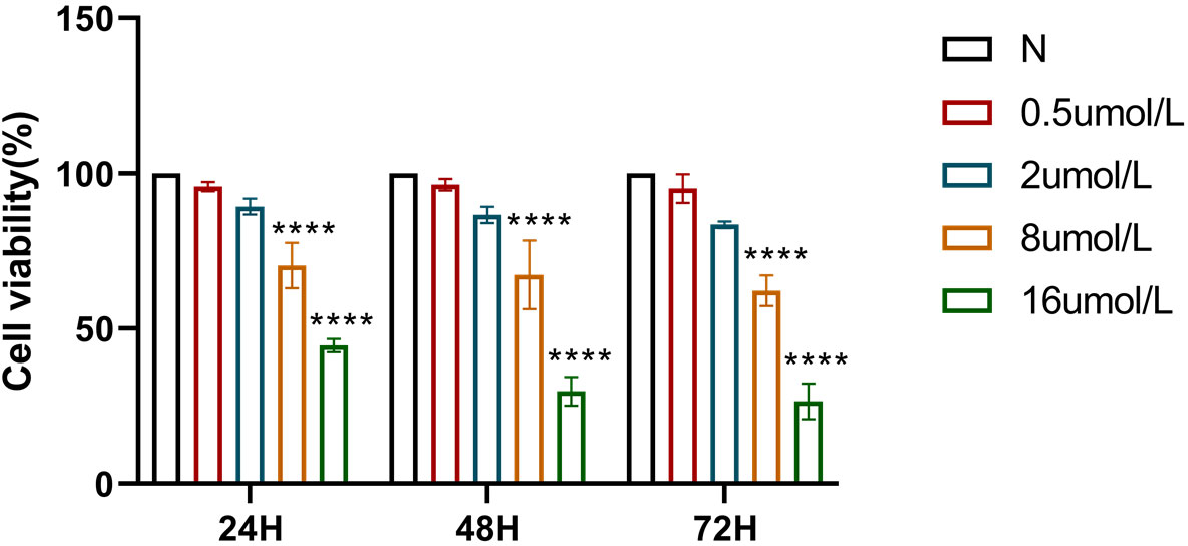
Effect of SRI on ARPE‐19 cell viability assessed by CCK‐8 assay. Cell viability was measured after treatment with different concentrations of SRI (0.5, 2, 8 and 16 μmol/L) for 24, 48 and 72 h under normal glucose conditions (5.5 mM). Data are presented as mean ± SEM (*n* = 3). ****p < 0.001 versus control group.

Treatment of ARPE‐19 cells with SRI (0.5, 2, 8 and 16 μmol/L) for 24, 48 and 72 h resulted in a dose‐ and time‐dependent reduction in cell viability, with statistically significant effects observed at concentrations ≥ 8 μmol/L (*p* < 0.05). Notably, at 72 h, both 8 and 16 μmol/L SRI markedly decreased cell viability compared with the control group. In contrast, at 24 h, only the 16 μmol/L treatment produced a significant reduction. Importantly, SRI at 2 μmol/L did not significantly affect cell viability at any of the time points examined. Therefore, 2 μmol/L was selected for all subsequent experiments. Unless otherwise specified, cells were treated with 25 mM glucose for 48 h before analysis (Figure 1).

### 3.2 Effect of SRI on the Wnt/β‐Catenin Pathway and VEGF Expression

**FIGURE 2:**
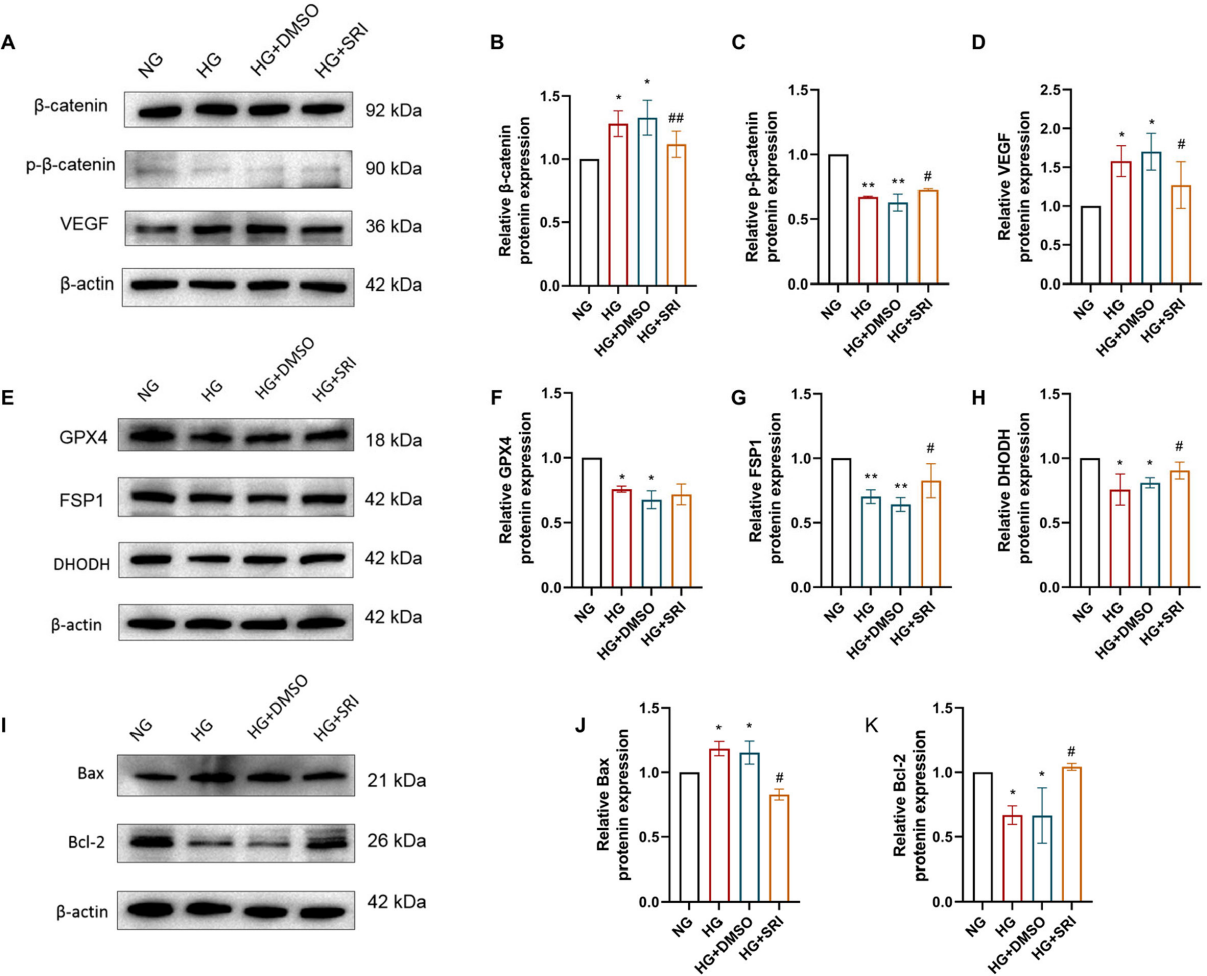
Western blot analysis of the effects of SRI on β‐catenin signalling, ferroptosis‐related proteins and apoptosis‐related proteins in high‐glucose‐induced ARPE‐19 cells. (A–D) Expression levels of β‐catenin, p‐β‐catenin and VEGF; (E–H) Expression of ferroptosis‐related proteins, including GPX4, DHODH and FSP1; (I–K) Expression of apoptosis‐related proteins Bax and Bcl‐2. Data are presented as mean ± SEM (*n* = 3). *p < 0.05, **p < 0.01 versus NG group; #*p* < 0.05, ##*p* < 0.01 versus HG group.

As shown in Figure 2A–D, the expression levels of β‐catenin and phosphorylated β‐catenin (p‐β‐catenin) differed significantly among the experimental groups (*p* < 0.05). Compared with the NG group (5.5 mM glucose), high glucose (HG, 25 mM glucose) exposure for 48 h markedly increased β‐catenin and VEGF expression while decreasing p‐β‐catenin levels (*p* < 0.05), indicating activation of the canonical Wnt/β‐catenin signalling pathway under hyperglycemic conditions. SRI treatment (HG + SRI) reversed these effects. Specifically, it significantly reduced β‐catenin and VEGF expression and increased p‐β‐catenin levels compared with the HG group (all *p* < 0.05). These results indicate that SRI inhibits Wnt/β‐catenin pathway activation and suppresses VEGF expression under high‐glucose conditions (Figure 2A–D).

### 3.3 Effects of SRI on Ferroptosis‐Related Protein Expression

As shown in Figure 2E–H, the expression of ferroptosis‐related proteins differed significantly among the experimental groups (*p* < 0.05). Compared with the NG group, high glucose (HG) exposure markedly reduced the expression of GPX4, DHODH and FSP1 in both the HG and HG + DMSO groups (*p* < 0.05). In contrast, treatment with SRI (HG + SRI) significantly increased the expression of DHODH and FSP1 relative to the HG group (both *p* < 0.05), whereas GPX4 levels remained unchanged (*p* > 0.05). Collectively, these findings suggest that SRI mitigates high‐glucose‐induced cellular damage, accompanied by preferential upregulation of the DHODH/FSP1 ferroptosis defence pathway, while the precise contribution of GPX4 requires further investigation (Figure 2E–H).

### 3.4 Effects of SRI on Apoptosis‐Related Proteins

The expression levels of apoptosis‐related proteins, including Bax and Bcl‐2, differed significantly among the experimental groups (*p* < 0.05). Specifically, compared with the NG group, Bax expression was markedly increased while Bcl‐2 expression was decreased in both the HG and HG + DMSO groups (*p* < 0.05). In contrast, treatment with SRI reversed these changes, as evidenced by reduced Bax expression and elevated Bcl‐2 levels in the HG + SRI group. Collectively, these findings suggest that SRI effectively attenuates high glucose‐induced apoptosis (Figure 2I–K).

### 3.5 SRI Attenuates High Glucose‐Induced Oxidative Stress in ARPE‐19 Cells

**FIGURE 3:**
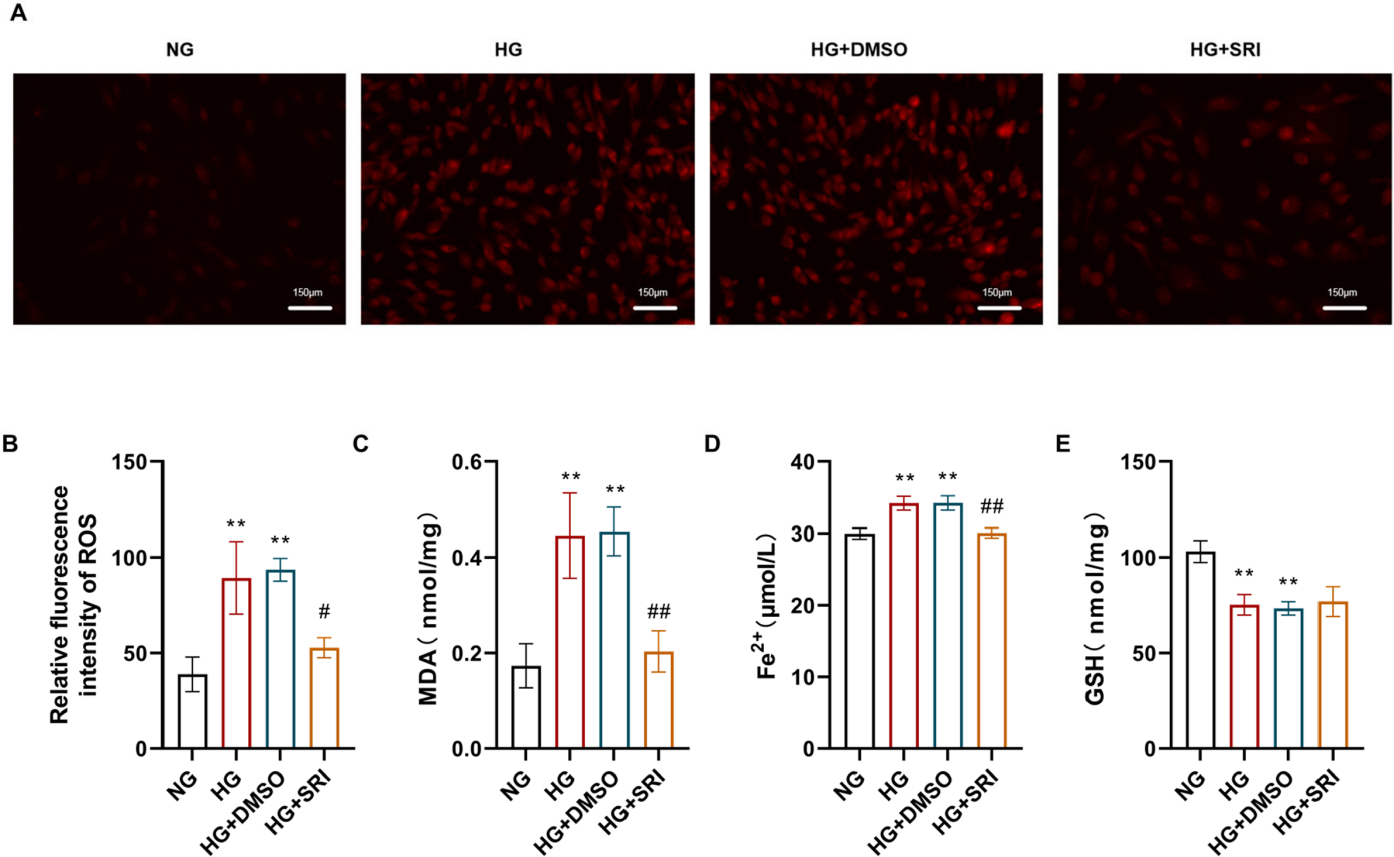
SRI attenuates oxidative stress and ferroptosis‐associated biochemical changes in high‐glucose‐treated ARPE‐19 cells. (A) Representative fluorescence images of intracellular ROS (scale bar = 150 μm). (B) Quantitative analysis of ROS fluorescence intensity. (C) Intracellular MDA levels. (D) Intracellular Fe^2+^ concentrations. (E) Intracellular GSH levels. Data are presented as mean ± SEM (*n* = 3). *p < 0.05, **p < 0.01 versus NG group; #*p* < 0.05, ##*p* < 0.01 versus HG group.

As shown in Figure 3, intracellular ROS, MDA, Fe^2+^ and GSH levels differed significantly among the four groups (NG, HG, HG + DMSO and HG + SRI) (*p* < 0.05). Compared with the NG group, high‐glucose exposure significantly increased ROS fluorescence intensity, MDA content and intracellular Fe^2+^ levels, while markedly decreasing GSH content in both the HG and HG + DMSO groups (*p* < 0.05 for ROS; *p* < 0.01 for MDA, Fe^2+^ and GSH). SRI treatment significantly reduced ROS fluorescence intensity, MDA content and Fe^2+^ levels compared with the HG and HG + DMSO groups (#*p* < 0.05, ##*p* < 0.01). In contrast, GSH levels were not significantly restored following SRI treatment. No significant differences were observed between the HG and HG + DMSO groups for any of these parameters, indicating that DMSO alone had no detectable effect. Overall, these results indicate that SRI attenuated high glucose‐induced oxidative stress by reducing ROS accumulation, lipid peroxidation and iron overload, although without significantly restoring intracellular GSH levels.

### 3.6 Effects of SRI on Mitochondrial Morphology

**FIGURE 4:**
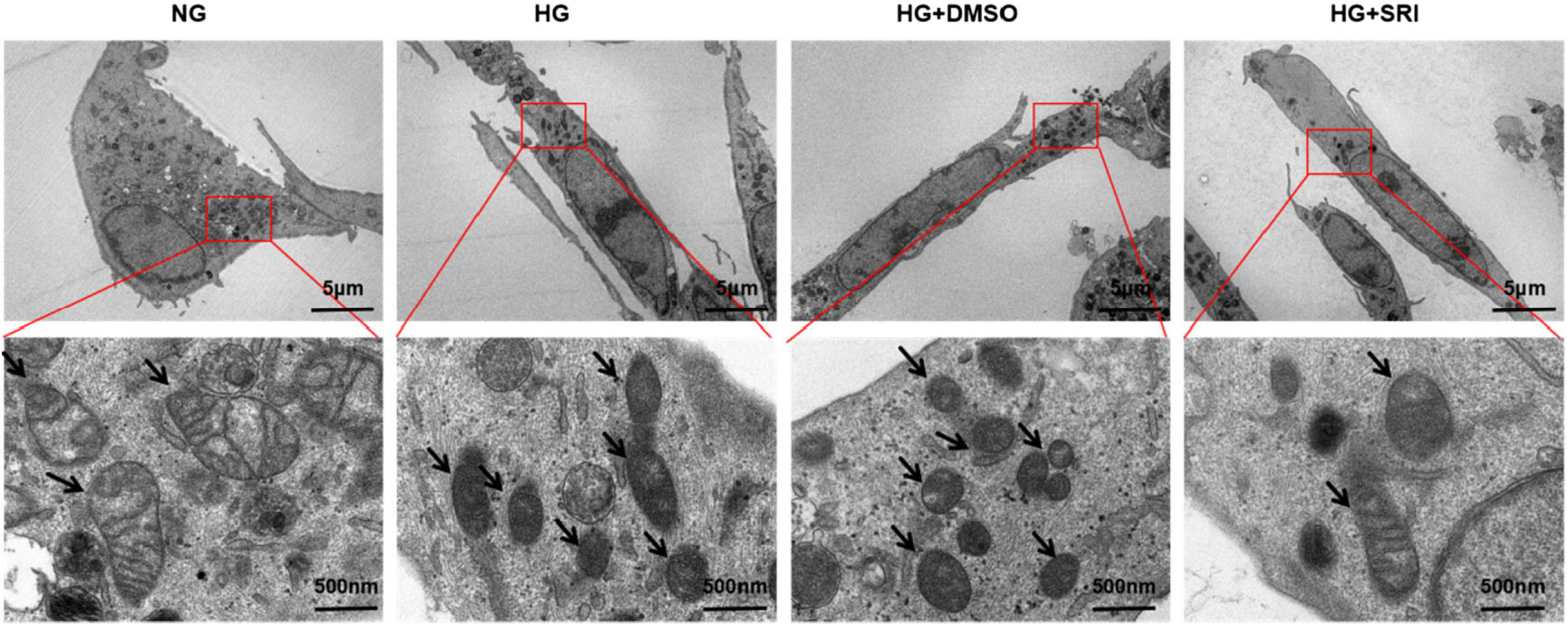
TEM images showing mitochondrial ultrastructural changes in ARPE‐19 cells. Cells were treated with 25 mM glucose for 48 h with or without SRI (2 μmol/L). NG: 5.5 mM glucose; HG: 25 mM glucose. Scale bars: Upper panel, 5 μm; lower panel, 500 nm. Arrows indicate representative mitochondrial abnormalities, including cristae loss, shrinkage and increased membrane density.

Transmission electron microscopy revealed distinct alterations in mitochondrial ultrastructure across the four experimental groups (Figure 4). In the NG group, mitochondria displayed typical elongated or oval shapes with well‐organised cristae and intact outer membranes, indicating preserved structural integrity. In contrast, both the HG and HG + DMSO groups exhibited severe mitochondrial damage, characterised by marked shrinkage, reduced volume, cristae disruption or loss, increased membrane electron density and occasional swelling or vacuolisation. Consistently, the mitochondrial morphology score was significantly elevated to 3.2 ± 0.2 compared with 1.2 ± 0.1 in the NG group (*p* < 0.001). SRI treatment markedly improved mitochondrial integrity, as evidenced by restored cristae organisation, reduced membrane electron density and an ultrastructural profile resembling that of the NG group. Accordingly, the morphology score decreased to 1.8 ± 0.2 (*p* < 0.01 vs. HG). Collectively, these findings indicate that SRI effectively attenuates high glucose–induced mitochondrial ultrastructural injury in ARPE‐19 cells.

## 4 Discussion

This study provides the first experimental evidence that the FZD7 inhibitor SRI protects ARPE‐19 cells against high glucose‐induced injury. To our knowledge, this is the first study to show that pharmacological inhibition of FZD7 protects RPE cells against ferroptosis and oxidative injury under hyperglycemic conditions. Although FZD7 has been linked to retinal angiogenesis, its role in RPE ferroptosis remains unknown. This protective effect is mechanistically linked to inhibition of the Wnt/β‐catenin signalling pathway, accompanied by reduced ferroptosis, apoptosis and OS. Together, these findings establish the FZD7/β‐catenin/ferroptosis axis as a key regulatory pathway in DR and highlight its therapeutic potential. CCK‐8 assays were conducted to determine appropriate experimental conditions. SRI decreased ARPE‐19 cell viability in a dose‐ and time‐dependent manner and a concentration of 2 μmol/L was selected based on the IC50 value and overall viability profiles. Under hyperglycemic conditions, SRI markedly reduced total β‐catenin levels while increasing p‐β‐catenin, confirming inhibition of Wnt/β‐catenin signalling. This result aligns with the canonical Wnt signalling paradigm, in which pathway activation stabilises β‐catenin and promotes its nuclear translocation. In contrast, FZD7 inhibition enhances β‐catenin phosphorylation and its subsequent ubiquitin–proteasome–mediated degradation, thereby limiting intracellular accumulation [11]. However, our conclusion regarding Wnt/β‐catenin pathway activation is currently based primarily on changes in total and phosphorylated β‐catenin protein levels. Direct assessment of β‐catenin nuclear translocation by immunofluorescence or immunohistochemistry would provide more definitive evidence for pathway activation and should be addressed in future studies.

Notably, Wnt/β‐catenin signalling exerts context‐dependent effects in DR. Under physiological conditions, it supports cellular homeostasis; however, its pathological overactivation promotes inflammation and cellular injury [25]. Consistent with this dual role, our results show that pathway inhibition alleviates hyperglycemia‐induced damage. This finding suggests that excessive Wnt/β‐catenin activation contributes to retinal injury, potentially through induction of ferroptosis. This interpretation is further supported by previous evidence demonstrating that iron chelators attenuate ferroptosis‐associated oxidative damage by suppressing Wnt/β‐catenin signalling in ARPE cells [26]. In addition to its involvement in ferroptosis, Wnt/β‐catenin signalling appears to regulate angiogenic responses. VEGF, a key mediator of vascular permeability and pathological neovascularisation, is a central therapeutic target in DR [27]. In this study, SRI not only inhibited Wnt/β‐catenin signalling but also reduced VEGF expression, indicating that the FZD7‐Wnt/β‐catenin axis may act upstream of VEGF transcription. Although VEGF upregulation under hyperglycemic conditions is commonly attributed to hypoxia‐inducible factor‐1α activation [28], our findings suggest that Wnt/β‐catenin signalling represents an alternative pathway linking metabolic stress to vascular dysfunction in DR.

Another key finding is that SRI selectively modulates ferroptosis‐related pathways. It upregulates the ferroptosis suppressors DHODH and FSP1, while GPX4 protein levels remain unchanged, indicating a GPX4‐independent mechanism. DHODH, located in the mitochondrial inner membrane, converts CoQ10 to CoQ10H_2_, a lipid‐soluble antioxidant that inhibits lipid peroxidation [16]. Likewise, FSP1 reduces CoQ10 through NAD(P)H‐dependent reactions. Together with DHODH, it forms a complementary ferroptosis defence system [17]. The simultaneous upregulation of DHODH and FSP1 suggests that SRI enhances mitochondrial antioxidant capacity and thereby limits ferroptotic damage. Consistent with this interpretation, GPX4, a canonical ferroptosis regulator that uses GSH to detoxify lipid peroxides [14], is not affected by SRI treatment. This finding indicates that the protective effects of SRI do not depend on the classical GPX4 pathway. Instead, increasing evidence shows that DHODH and FSP1 function as parallel and compensatory systems that maintain redox homeostasis when GPX4 activity is insufficient [29]. In line with this model, our results suggest that SRI preferentially activates the DHODH/FSP1 axis to counteract ferroptosis in RPE cells. Ultrastructural analysis further revealed improved mitochondrial morphology after SRI treatment. This observation supports its role in ferroptosis inhibition, as mitochondrial shrinkage, cristae loss and increased membrane density are hallmark features of ferroptosis [12].

In addition to its effects on ferroptosis, SRI also modulates apoptosis‐related signalling. It regulates the expression of Bax and Bcl‐2, two key mitochondrial apoptosis regulators, thereby counteracting high glucose‐induced apoptotic damage. As the Bax/Bcl‐2 ratio is a critical determinant of apoptotic susceptibility, the decrease in Bax coupled with the increase in Bcl‐2 indicates inhibition of mitochondria‐mediated apoptosis. Apoptosis and ferroptosis are mechanistically interconnected rather than independent processes. Mitochondrial dysfunction, for example, is both a hallmark of ferroptosis and a trigger of apoptosis [30, 31]. In this context, SRI‐mediated preservation of mitochondrial integrity may concurrently suppress both cell death pathways. Consistent with this, SRI reduces intracellular Fe^2+^, ROS and MDA, supporting its antioxidative capacity. Given that iron serves as a key catalyst in ferroptosis [32], its reduction may partly result from SRI‐mediated regulation of iron metabolism‐related proteins, such as ferritin and transferrin receptors.

Despite these promising findings, several limitations should be acknowledged. Although our data demonstrate that SRI modulates multiple ferroptosis‐related biomarkers and ameliorates ferroptosis‐associated mitochondrial abnormalities [33], these findings are consistent with ferroptotic stress but do not constitute direct functional evidence of ferroptosis. Therefore, the inclusion of a rescue experiment using a specific ferroptosis inhibitor, such as Ferrostatin‐1 [34], would provide additional mechanistic validation and further strengthen the causal link between SRI treatment and ferroptosis inhibition [35]. More importantly, although our findings are consistent with ferroptotic stress, direct functional evidence of ferroptosis is still lacking. Future studies using ferroptosis‐specific inducers and inhibitors are needed to further validate this conclusion. This remains an important direction for future investigation [36]. As an in vitro study, the present work cannot fully recapitulate the complex pathological microenvironment of diabetic retinopathy [37]. In particular, it does not capture the dysfunction of the iBRB, involving endothelial cells, pericytes and glial cells or the integrity of the oBRB, which is maintained by RPE cells. Nor does it reflect the dynamic interactions between these two barrier systems and retinal glial cells [38]. We also acknowledge the absence of an osmotic control group (e.g., mannitol) in the current study. Although the high‐glucose ARPE‐19 model has been widely validated and the protective effect of SRI supports the involvement of glucose‐specific pathogenic mechanisms, the contribution of osmotic stress cannot be entirely excluded [24, 26]. Future studies incorporating appropriate osmotic controls will be necessary to further validate the specificity of the observed effects. Finally, the current findings are derived exclusively from an in vitro cell culture model and therefore cannot establish the physiological relevance of the proposed mechanism in vivo. Although ARPE‐19 cells cultured under high‐glucose conditions represent a well‐established model for investigating RPE injury in diabetic retinopathy, they cannot fully recapitulate the complexity of the retinal microenvironment. Therefore, validation in appropriate animal models of diabetic retinopathy and, where feasible, in clinical samples will be necessary to establish the physiological relevance and translational potential of targeting FZD7. These studies represent important directions for future investigation.

In summary, this study shows that the FZD7 inhibitor SRI alleviates hyperglycemia‐induced ferroptosis, apoptosis and oxidative stress in ARPE‐19 cells by inhibiting Wnt/β‐catenin signalling. Mechanistically, our findings suggest that the protective effects of SRI are associated with suppression of Wnt/β‐catenin signalling and preferential activation of the GPX4‐independent ferroptosis defence proteins DHODH and FSP1, accompanied by preservation of mitochondrial integrity. Although the precise contribution of GPX4 and the causal role of ferroptosis require further validation, these findings support the involvement of the FZD7/β‐catenin/ferroptosis axis in RPE cell injury during diabetic retinopathy and highlight FZD7 as a promising therapeutic target for limiting retinal damage.

## Author Contributions

**Jiaojiao Jiang:** conceptualization, writing – original draft, writing – review and editing, visualization, validation, methodology, software, formal analysis, data curation. **Zhixiang Ding:** writing – review and editing, funding acquisition, conceptualization, project administration, supervision, resources. **Liwu Tan:** investigation, visualization, validation, software, formal analysis, data curation. **Liu Zheng:** investigation, validation, visualization, software, methodology.

## Funding

This study was funded by the National Natural Science Foundation of China (Grant 82160197); Graduate Research Program of Guilin Medical University (Grant GYBK2025003).

## Ethics Statement

The authors have nothing to report.

## Consent

The authors have nothing to report.

## Conflicts of Interest

The authors declare no conflicts of interest.

## Data Availability

The data that support the findings of this study are available from the corresponding author upon reasonable request.
